# Testing the Limits of Morphology: A Comprehensive Morphometric Study of the Sister Lineages *Lasiocyano* Galleti‐Lima, Hamilton, Borges and Guadanucci, 2023 and *Lasiodora* C. L. Koch, 1850 (Theraphosidae, Mygalomorphae)

**DOI:** 10.1002/jmor.70123

**Published:** 2026-04-12

**Authors:** Analice G. Marquezin‐Gomes, Arthur Galleti‐Lima, Millke J. A. Morales, José Paulo L. Guadanucci

**Affiliations:** ^1^ Programa de Pós Graduação em Zoologia, Instituto de Biociências Universidade Estadual Paulista (UNESP) Botucatu São Paulo Brazil; ^2^ Laboratório de Aracnologia de Rio Claro Universidade Estadual Paulista (UNESP) Rio Claro São Paulo Brazil; ^3^ Laboratório de Coleções Zoológicas Instituto Butantan São Paulo São Paulo Brazil; ^4^ Departamento de Biodiversidade Universidade Estadual Paulista (UNESP) Rio Claro São Paulo Brazil

**Keywords:** geometric morphometrics, morphological conservatism, Neotropical region, tarantulas, Theraphosinae

## Abstract

Morphological conservatism and homoplasy pose significant challenges for the systematics of mygalomorph spiders, limiting the number of reliable morphological characters available for species identification, particularly in Theraphosidae. Closely related taxa frequently display high phenotypic similarity, which limits the resolution of morphology‐based approaches. In this study, we conducted the most extensive morphometric analysis to date within Theraphosidae, with the objective of explicitly testing how much morphological information is retained within the *Lasiocyano sazimai* and *Lasiodora* lineage. We applied a morphometric framework combining linear morphometry and geometric morphometry, including multivariate statistics, discriminant analyses, and cross‐validation, to evaluate interspecific differentiation in this group. Although some analyses recovered statistically significant differences among taxa, extensive morphological overlap, high intraspecific variation, and pronounced overfitting in cross‐validated classifications consistently reduced the discriminatory power of the methods. As a result, none of the approaches provided reliable diagnostic separation between *Lasiocyano sazimai* and *Lasiodora* species. Our results indicate that morphology alone reaches clear limits of resolution within this lineage. The only characters that remained consistently informative for distinguishing the genera were the presence of stridulatory setae in *Lasiodora* and the distinctive blue‐purplish setae of *Lasiocyano*. By explicitly testing the limits of morphometric and morphological inference in a morphologically conservative group, this study helps clarify the actual scope and limitations of morphology‐based systematics in Theraphosidae.

## Graphical abstract

An extensive morphometric framework combining linear and geometric morphometry was applied to evaluate interspecific differentiation within the Lasiocyano sazimai and Lasiodora lineage (Theraphosidae). Despite statistically significant differences in some analyses, extensive morphological overlap and high intraspecific variation consistently reduced the discriminatory power of morphometric methods. These results reveal clear limits of morphology‐based systematics in this morphologically conservative group.

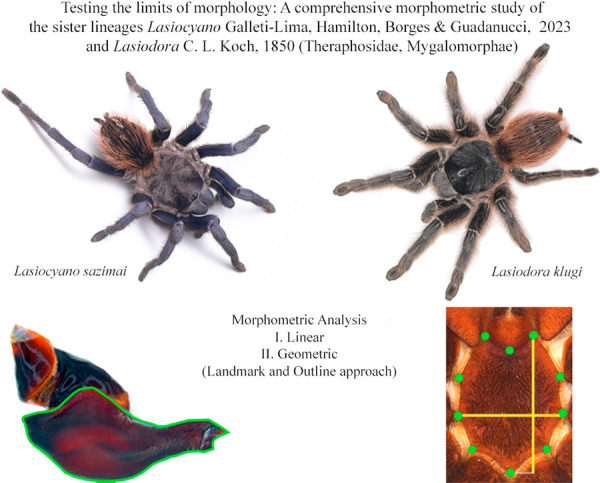

## 1 Introduction

The monophyletic infraorder Mygalomorphae comprises 32 families including sheet‐web spiders, trapdoor spiders and tarantulas (Raven 1985; Bond et al. 2012; Hedin et al. 2018; Godwin et al. 2018; Hedin et al. 2019; Opatova et al. 2020; Montes de Oca et al. 2022; Perafán et al. 2025; World Spider Catalog 2025). Mygalomorph spiders are characterized by similar life history, behavioral and morphological traits (Bond and Opell 2002). From an evolutionary perspective, these spiders exhibit relatively conservative and frequently homoplastic morphological patterns, resulting in a limited number of informative characters for systematics, making the exclusive use of traditional morphological traits problematic, often leading to an underestimation of species diversity (Goloboff 1995; Bertani 2001; Hedin and Bond 2006; Pérez‐Miles and Perafán 2017). To address these limitations, alternative taxonomic approaches have been applied in some mygalomorph families, particularly through the use of continuous characters and morphometric methodologies, including geometric morphometrics (Korba et al. 2022; Colpani‐Sartori and Guadanucci 2024; Sagastume‐Espinoza et al. 2024; Groppo et al. 2025), linear morphometrics (Hamilton et al. 2016) and studies integrating both approaches (Fonseca‐Ferreira et al. 2023; Moeller et al. 2024).

**Figure 1:**
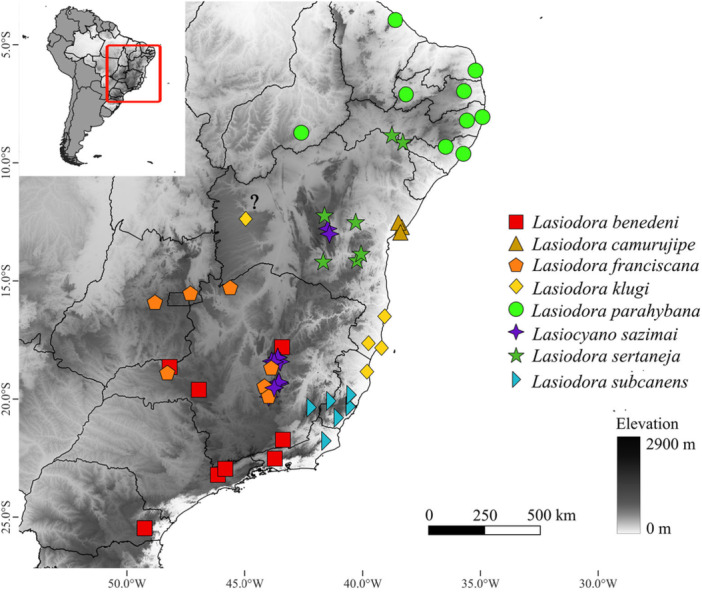
Distribution map of *Lasiocyano sazimai* and *Lasiodora* species. (?) represents questionable record.

In the Neotropical region, Theraphosidae Thorell, 1869 is mostly represented by subfamily Theraphosinae, which are specially characterized for its urticating setae on the dorsal abdomen (Bertani 2001; Bertani and Guadanucci 2013). Among Theraphosinae, the monotypic genus *Lasiocyano* Galleti‐Lima et al. 2023 is endemic to highlands of “campos rupestres” phytophysiognomy in the Brazilian Espinhaço mountain range. *Lasiocyano sazimai* (Bertani et al. 2011) has a restricted distribution in two main populations: one in the Espinhaço Meridional population (southern Espinhaço; Minas Gerais state) and other in Chapada Diamantina (northern Espinhaço; Bahia state) (Figure 1). Characterized by its large size and the presence of blue‐purplish setae covering chelicerae, carapace and legs, *Lasiocyano sazimai* is a threatened species since 2014 by the National list of threatened species of Brazil (ICMBio 2018). This conservation status reflects both illegal trade of exotic pets and the degradation of the “campos rupestres” habitat, combined with restricted distribution (ICMBio 2018; Marshall et al. 2022). This species was originally described under Pterinopelma Pocock, 1901 by Bertani et al. (2011), with a misidentification of the male, later corrected by Bertani and Leal (2016). In the same publication, the authors also proposed that *P. sazimai* should not belong to genus *Pterinopelma*, not even to other related genus, as *Lasiodora* C. L. Koch, 1850, Nhandu Lucas, 1983 and Vitalius Lucas, Silva and Bertani 1993, so they decided to not propose a formal transfer. Later, this hypothesis was corroborated by Galleti‐Lima and Guadanucci (2018) in a study of stridulatory setae of Theraphosinae, which recovered *Lasiocyano sazimai* as sister group of *Nhandu* and related to *Vitalius*. Most recently, Galleti‐Lima et al. (2023) conducted a phylogenomic study on Lasiodoriforms spiders in which *P. sazimai* was recovered as sister taxa of Lasiodora Koch, 1850 in a well‐supported clade, with *Parvicarina felipeleitei* (Bertani and Leal 2016), as sister group of *Lasiocyano sazimai* + *Lasiodora*. These transferences were based on the distinct and exclusive characters present in these genera, as the presence of stridulatory setae in the coxae I and II above its suture in *Lasiodora*, poor‐developed keels in the male palpal bulb of *Parvicarina* and presence of blue‐purplish setae of *Lasiocyano*.

**Figure 2:**
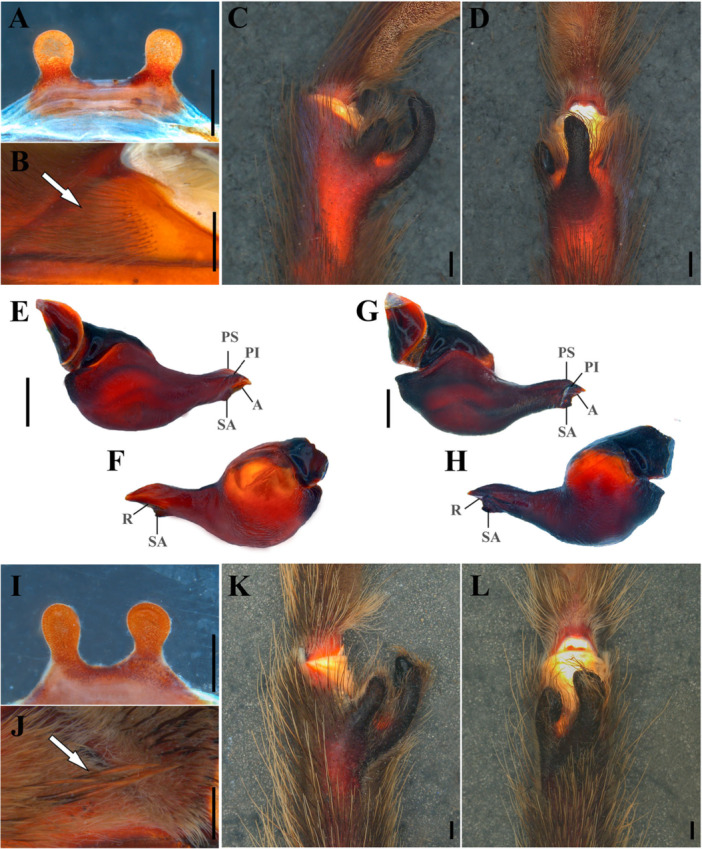
Morphological characters of *Lasiocyano sazimai* (A–F) and *Lasiodora klugi* (G–L). (A, I) spermathecae (UFMG17202A; IBSP106368). (B, J) stridulatory apparatus on the prolateral coxae I (CAD466; CAD1524); plumose (B, J) and velvet (J) striduling setae are indicated by white arrows. (C, K) bifid tibial apophysis, prolateral view. (D, L) tibial apophysis and metatarsus I, ventral view (UFMG20075; CAD1527). (E, G) male palpal bulb, prolateral view. (F, H) male palpal bulb, retrolateral view (UFMG23464; IBSP106537). (E–H) palpal bulb keels: A, apical; PI, prolateral inferior; PS, prolateral superior; R, retrolateral; SA, subapical. Scale bar: 1 mm.

**Figure 3:**
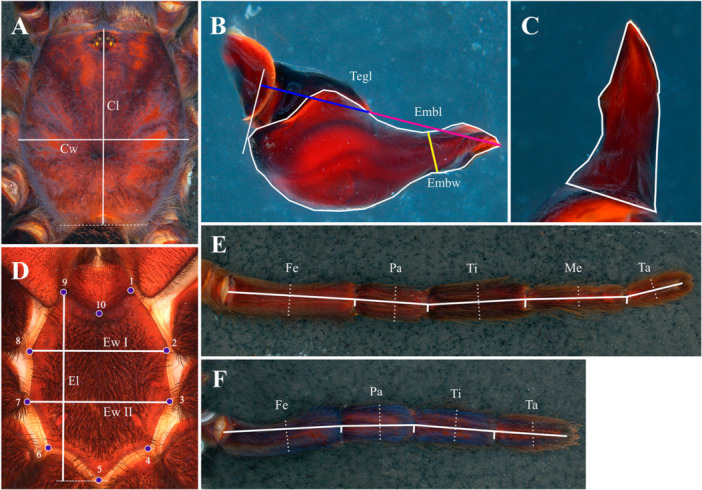
Morphometrical traits analysed and their measurements. (A) Carapace length (Cl) and width (Cw). (B) Male palpal bulb, prolateral view, embolus length (Embl—pink line) and width (Embw—yellow line), and tegulum length (Tegl—blue line); outline. (C) Male palpal bulb embolus dorsal view; outline. (D) Sternum length (El) and width (EwI: 2–8 interlandmark distance; EwII: 3–7 interlandmark distance); landmarks positions. (E) Leg dorsal view. (F) Pedipalp dorsal view. (E–F) length (continuous line) and width (dotted line); articles femur (Fe), patella (Pa), tibia (Ti), metatarsus (Me) and tarsus (Ta).

The morphological similarity between *Lasiocyano* and *Lasiodora* is well documented in previous systematic studies. As in some other genera of Lasiodoriforms, males of *Lasiocyano* and *Lasiodora* can be identified by their bifid tibial apophysis (Figure 2C,K), metatarsus I touching the retrolateral branch of the tibial apophysis when flexed (Figure 2D,L), presence of a subapical and retrolateral keel, and the triangular shape of the subapical keel in the palpal bulb and its embolus apex slightly laterally flattened (Bertani 2001; Bertani and Leal 2016; Bertani 2023 (Figure 2E,G). Females of these genera present the combination of: short spermathecal receptacles separated by a fused and sclerotized area (Figure 2A,I); and presence of urticating setae of types I and III on the dorsal surface of the abdomen (Bertani et al. 2011; Bertani 2023). The only consistently reported morphological characters that distinguish these genera are the presence of stridulatory apparatus with plumose setae on the prolateral coxae I‐IV in *Lasiodora* (Figure 2B,J), and additionally the iridescent blue‐purplish setae in *Lasiocyano* (Figure 3A) (Bertani et al. 2011; Bertani and Leal 2016; Galleti‐Lima and Guadanucci 2019; Galleti‐Lima et al. 2023; Bertani 2023).

The high morphological similarity between *Lasiocyano* and *Lasiodora* provides an exceptional opportunity to explicitly test the limits of morphological inference within a taxonomically problematic lineage. Given their close phylogenetic relationship and the broadly similar life‐history strategies, a certain degree of phenotypic resemblance between these two genera is not unexpected (Wilson et al. 2023). However, the extent to which morphology retains informative value within this lineage remains unclear. In this context, the long‐recognized difficulty of establishing reliable diagnostic characters in Theraphosidae, described by Raven (1990) as a “taxonomical and nomenclatural nightmare”, motivates a quantitative assessment of morphological variation in the *Lasiocyano+Lasiodora* clade. In the present study, we explicitly evaluate the extent to which morphology retains informative value within a lineage known for strong morphological conservatism, by applying the most extensive morphometric framework implemented for Theraphosidae to date. This framework integrates linear morphometry of legs, pedipalps, carapace, sternum and male palpal bulb; geometric morphometry, including landmark‐based analyses of the sternum and outline‐based analyses of the male prolateral palpal bulb and the dorsal view of its embolus.

## 2 Material and Methods

### 2.1 Material

Material examined is deposited in the following collections and their respective curators: CAD = Coleção Aracnológica Diamantina, Rio Claro, São Paulo, Brazil (J. P. L. Guadanucci); IBSP = Instituto Butantan, São Paulo, São Paulo, Brazil (A. D. Brescovit); UFMG = Centro de Coleções Taxonômicas, Universidade Federal de Minas Gerais, Belo Horizonte, Brazil (A. J. Santos). The species analysed were *Lasiocyano sazimai* (*n* = 35), *Lasiodora benedeni* (*n* = 10), *Lasiodora camurujipe* (*n* = 4), *Lasiodora franciscana* (*n* = 9), *Lasiodora klugi* (*n* = 10), *Lasiodora parahybana* (*n* = 10), *Lasiodora sertaneja* (*n* = 14) and *Lasiodora subcanens* (*n* = 10). Specimens were identified based on Bertani et al. (2011), Bertani and Leal (2016), Galleti‐Lima et al. (2023) and Bertani (2023). Immature individuals and those with damaged structures were excluded from analyses. Males and females specimens were analysed separately in all analyses.

### 2.2 Image Acquisition

Photographs and measurements of the sternum (ventral view), spermathecae (ventral view), palpal bulb (prolateral view) and palpal embolus (dorsal view) were taken with a Leica MC170 digital camera mounted on a Leica M205C stereomicroscope using LAS Core software v.4.12.0. All specimens were immersed in 70% ethanol and palpal bulbs and spermathecae were immersed in ethanol gel to stabilize their position under the microscope. Photographs were obtained at the Laboratório de Aracnologia de Rio Claro and the Laboratório de Biodiversidade e Sistemática de Insetos, Universidade Estadual Paulista (UNESP), campus Rio Claro, São Paulo, Brazil.

### 2.3 Linear Morphometric Analysis

Linear measurements of sternum and male palpal bulb were obtained under the stereomicroscope using LAS Core software. Length and width measurements of carapace, palps and leg articles (femur, patella, tibia, metatarsus, tarsus/cymbium) were taken with a caliper, as these structures exceeded the size limits of the stereomicroscope. Measurements of the male palpal bulb were taken using the LAS Core. In total, 34 relative measurements were generated from the ratios of length and width of carapace, sternum length relative to the width between landmarks 2 and 8, and 3 and 7 (Table 1), length and width of articles of the palp and legs I–IV, total length of the appendages relative to carapace width, length by the width of embolus and length of embolus relative to tegulum length (Figure 3).

**Table 1:** Type and description of sternum landmarks used in geometric morphometric analysis of *Lasiocyano* and *Lasiodora*.

Landmark	Type	Description
1	I	Insertion of left endite and labium
2	I	Insertion area of left coxae I
3	I	Insertion area of left coxae II
4	I	Insertion area of left coxae III
5	I	Insertion area of left and right coxae IV
6	I	Insertion area of right coxae III
7	I	Insertion area of right coxae II
8	I	Insertion area of right coxae I
9	I	Insertion area of the right endite/basal right extremity of the labium
10	II	Maximum median basal curvature of the labium

The measurements of the male palpal bulb embolus and tegulum (Figure 3B) were based on the ones suggested by Bertani (2023). Although the tegulum and embolus of the male mygalomorph palpal bulb are fused, which makes delimiting these structures difficult, the author proposes that the embolus length is “measured from its apex to the subtegulum” (pink line), the tegulum length (blue line) is defined by “a perpendicular line projected from the tegulum posterior edge” (white line) and the embolus width is measured “at the half of the embolus length” (yellow line). In Bertani (2023), these ratios between the embolus length and width (Character 0; Bertani 2023) and the embolus length relative to the tegulum length (Character 1; Bertani 2023) (Figure 3B) were considered as continuous characters. Ratios were used to minimize size‐related variation and to describe proportional morphological variation among species and genera (Fonseca‐Ferreira et al. 2023).

Analyses were conducted with PAST software 5.2.1. (Hammer et al. 2001). Variables were tested for normality (Shapiro–Wilk) and correlation (Spearman's *ρ*). To reduce redundancy, pairwise correlations were examined and when two or more variables were strongly correlated, a single representative variable was retained to capture the main source of variation. Other variables were excluded from further analyses (see Supporting Information 02). The Principal Coordinates Analysis (PCoA) was performed using Gower similarity coefficient. To compare groups, a Discriminant Analysis (DA) was conducted to maximize differences between species groups and visualize morphological structuring between species. A confusion matrix was generated in DA to analyse the accuracy of groups prediction with and without cross‐validation (jackknifed). The DA biplot was applied to visualize the sample distribution along discriminant component axes and the contribution of each variable to the observed pattern. Finally, a one‐way PERMANOVA (Gower similarity index; 10,000 permutations) was performed to test the significance of differences among groups (*p*‐value < 0.05).

Additionally, ratios between embolus length and width, and between embolus length and tegulum length were tested to evaluate differences between *Lasiocyano sazimai* and the species of *Lasiodora*, as these ratios showed higher values for *Lasiocyano sazimai* compared to *Lasiodora* in the morphological matrix of Bertani (2023). Comparative statistical analysis was performed in R Studio software v. 2025.5.1.513 (R Core Team 2025). The normality of both ratio variables was assessed using the Shapiro–Wilk test (“shapiro.test”). The assumption of homogeneity of variance was evaluated with Levene's test (“leveneTest”, package car; Fox and Weisberg 2019), and a one‐way Analysis of Variance (ANOVA) (“aov”) was performed to compare the means of ratios of the male palpal bulb of *Lasiocyano* and *Lasiodora* species. Boxplots and density distribution plots were generated to visualize the data (package ggplot2; Wickham 2016). Mean and median of each species were calculated with package dplyr (Wickham et al. 2025).

### 2.4 Geometric Morphometric Analysis

#### 2.4.1 Landmarks‐Based Analysis

Photographs of the sternum were converted to TPS format using TPSUtil software and landmarks were digitized with TPSdig2 (Rohlf 2015). A total of 10 landmarks were placed on the sternum in the order described in Table 1 (Moeller et al. 2024) (Figure 3D). Analyses were performed in MorphoJ 1.08.02 software (Klingenberg 2011). A Procrustes fit was performed to remove variation of size, position, and orientation, followed by the generation of a Covariant Matrix. Based on this matrix, a Principal Component Analysis (PCA) was conducted to identify the first axes of shape variation and visualize the distribution of the sternum landmarks in morphospace. To visualize the separation among groups and pairs of species, we performed a Canonical Variate Analysis (CVA) and Discriminant Function analysis (DFA) with cross‐validation and 1000 permutations. Significance of CVA results was assessed using global tests against the null hypothesis (Goodall's F, Pillai's trace; *p* < 0.05). In DFA, the significance was assessed examining the *p*‐values of the Mahalanobis test and the Procrustes test, and confusion matrix tables, based on the discriminant function and the cross‐validation. To correct allometry effects, a regression analysis was performed between the centroid size (independent variable) and the Procrustes coordinates (dependent variable). If significant for allometry, residuals of regression were used to generate a new Covariance Matrix and reperform the multivariate analysis. Mean shapes of the sternum in each analysis are represented by a dark blue shape (“positive” mean shape) and light blue shape (“negative” mean shape).

#### 2.4.2 Outlines‐Based Analysis

Outlines of the prolateral view of palpal bulb and dorsal view of the embolus (Figure 3B,C) were produced using GIMP software (GNU Image Manipulation Program; GIMP 2025). Outliness were traced from grayscale photographs with “vector” function. For the elliptic Fourier analysis, the prolateral view encompassed both tegulum and embolus as a single continuous outline, and the dorsal face of the embolus, comprehence its distal apex to the basal region, where its limits with the tegulum. In dorsal view of the male palpal bulb, the tegulum is visible as a rounded structure, from which the embolus appears to originate. Because these structures are fused and lack a clear morphological boundary, the delimitation of the embolus base is necessarily operational. In the present study, this boundary was defined by a straight reference line positioned across the transitional region between these structures, at the point where the rounded contour of the tegulum appears to terminate and the embolus shaft begins. The application of outline‐based morphometric analyses to the dorsal view of the embolus is relatively novel. This approach was adopted following the diagnostic criteria proposed by Bertani (2023), who demonstrated that the shape of the embolus apex in dorsal view provides reliable characters for distinguishing species of *Lasiodora*.

Subsequent multivariate analyses were performed in R Studio software (R Core Team 2025). Elliptic Fourier Analysis was conducted with the package Momocs v.1.3.0 (Bonhomme et al. 2014) and additional customized functions. The function “calibrate_harmonic_power_efourier” was used to determine the number of harmonics required to capture 95%–99% of shape variation. Once the harmonics number was defined, the function “eFourier” was applied to normalize rotation, size, and orientation. The PCA was performed to visualize the shape variation at the morphospace. The function “propVar” calculated the number of dimensions and their contributions (Principal Components—PCs), while “PContrib” calculates and plots the shape variation along Principal Component axes. Function “tps_iso” was used to visualize the variation of outlines between the extreme points (individuals) of the positive and negative portion of the Principal Component 1 in PCA. Discriminant Analyses (LDA) was performed to maximize groups variation with the functions “lda” and “lda_hist” from package MASS (Venables and Ripley 2002). Outputs included the contributions of the Linear Discriminants axes (LDs; proportion trace) and the confusion matrix. The PERMANOVA test was performed with the package “vegan” (function “adonis2”).

## 3 Results

### 3.1 Linear‐Based Results

**Figure 4:**
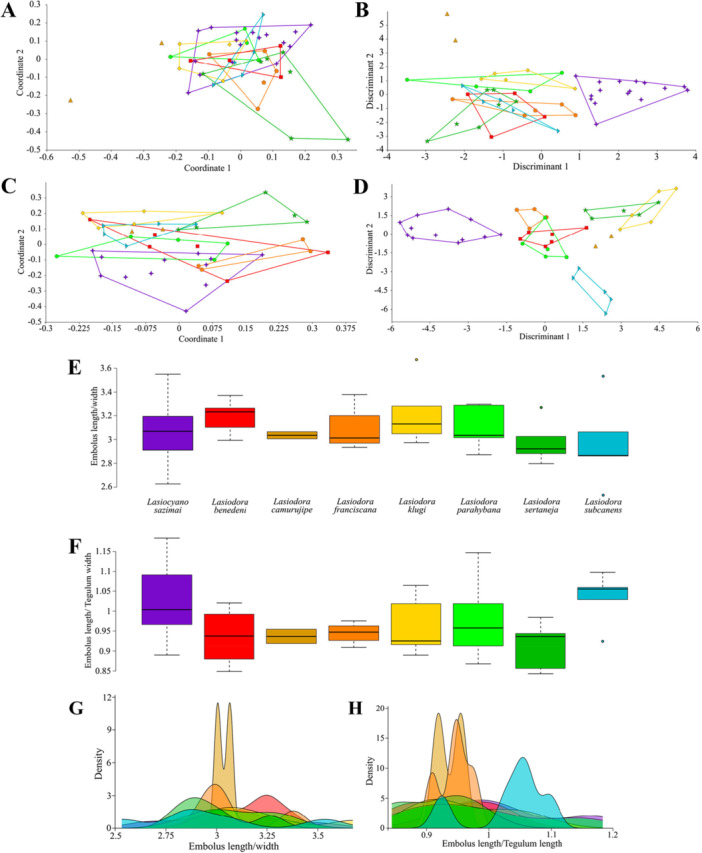
Linear‐based approaches results. (A, C) Principal Coordinates Analysis (PCoA) of *Lasiocyano* and *Lasiodora* females (A) and males (C). Discriminant analysis (DA), females (B) and males (D). (E–F) Boxplots of the ratios of the Embolus length and width (E), and the Embolus length/Tegulum length (F). (G–H) Density distribution of the ratios of the male palpal bulb. Colors and symbols for each species are the same as those used in Figure 1.

The PCoA recovered 46.02% (PC1 = 36.22%; PC2 = 9.8%) for female dataset (Figure 4A), and 37.96% (PC1 = 28.54%; PC2 = 9.42%) for male data set (Figure 4C). Both scatterplots showed overlaps between all species. Female DA presented 71.73% of variation in the two first axes (Discriminant 1 = 42.72%; Discriminant 2 = 29.01%) (Figure 4B). In the scatterplot, *Lasiodora sazimai* was separated from other *Lasiodora* species in the positive portion of Discriminant 1. In DA biplot, the leg II patella ratio was the only variable related with *Lasiocyano sazimai* position along the first and second axis (Supporting Information 3). The PERMANOVA test returned a significant *p*‐value (< 0.05) among *Lasiocyano sazimai* and *Lasiodora subcanens* (*p* = 0.0389), *Lasiodora sertaneja* (*p* = 0.008) and *Lasiodora camurujipe* (*p* = 0.005). Confusion matrix of DA recorded 77.08% of accuracy to predict the sample species, but after jackknifed the accuracy reduced to 37,5% (Supporting Information 04). The male's DA explained 72.3% of the variation (Discriminant 1 = 50.39%; Discriminant 2 = 21.91%) (Figure 4D), with *Lasiodora sazimai* grouped at the negative portion of Discriminant 1 axis. DA biplot indicated that ratios of carapace, pedipalp total length, pedipal tarsus and femur ratios, leg II patella and leg IV tarsus was related with *Lasiocyano sazimai* position in the scatterplot (Supporting Information 3). The confusion matrix presented 95.45% of accuracy and only 36,36% after cross‐validated. PERMANOVA confirmed significant differences between *Lasiocyano sazimai* and *Lasiodora franciscana* (*p* = 0.022), *Lasiodora klugi* (*p* = 0.005), *Lasiodora subcanens* (*p* = 0.032) and *Lasiodora sertaneja* (*p* = 0.0009) (Supporting Information 04).

In the comparative analysis of the male palpal bulb, ratios of embolus length and width, and of embolus length and tegulum length presented a normal distribution according to the Shapiro–Wilk test (*p* ≥ 0.05). Variances were homogeneous in Levene's test and ANOVA indicated no statistically significant differences in the mean ratios among species. The overlap between the interquartile ranges (IQR) in boxplots and similar median values support the similarity of these ratios among species (Figure 4E,F). Complementary, the density distribution exhibit broad overlap among species, and the low density and high dispersion of values reinforces the non‐significant ANOVA results and the similarity of the ratios (Figure 4G,H) (Supporting Information 05).

### 3.2 Landmark‐Based Results

**Figure 5:**
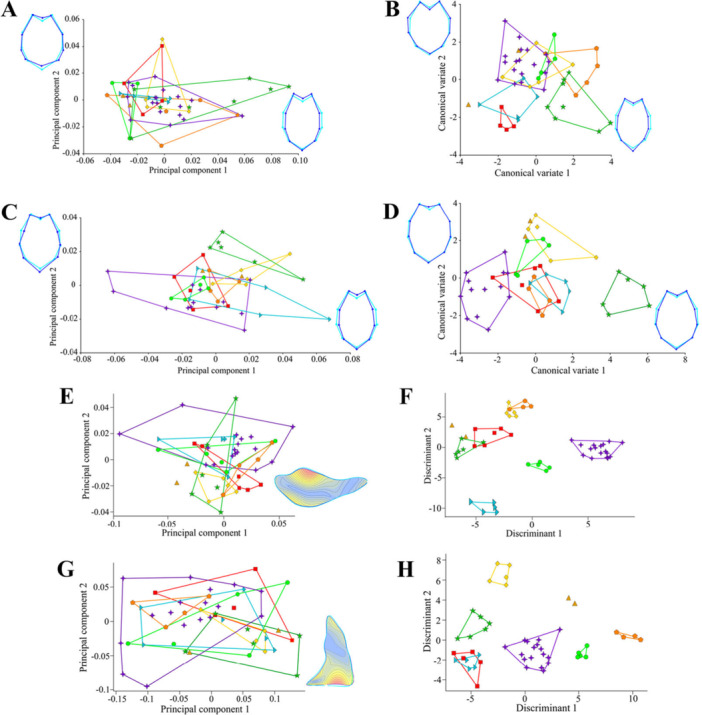
Geometric‐based approaches results. (A, C) Principal Component Analysis (PCA) of *Lasiocyano* and *Lasiodora*. (B, D) Canonical Variate Analysis (CVA). (A, B) Females; (C, D) males. The sided shapes represent the mean shape of sternum landmarks variation among the positive (darkblue lines and dots) and the negative (lightblue lines and circles) portion of canonical variate 1 (right shape) and 2 (left shape). (E, G) Principal component analysis (PCA) of *Lasiocyano* and *Lasiodora* male palpal bulb in outline approaches. The sided images represent the outline variation between the extreme shapes of PC1, with warmer colors indicating regions of greater shape variation. (F, H) Linear Discriminant analysis (LDA) of outline approaches. (E, F) prolateral view; (G, H) dorsal embolus view. Colors and symbols for each species are the same as those used in Figure 1.

The linear regression analysis indicated a significant allometric effect of 12.9% (*p*‐value < 0.05) only for females; therefore, subsequent analysis was performed with regression residuals. For females, landmark‐based PCA resulted in 16 principal components, with the first two principal components (PCs) explaining 63.5% of total shape variation (PC1 = 51.9%; PC2 = 11.6%). Variation along PC1 is associated with a more elongated and narrower sternum at the positive extreme of the axis, reflected by the inward position of landmark pairs 2–8 and 3–7 and the outward position of landmark 10 relative to the negative extreme. Along PC2, the sternum shapes at both extremes remain overall rounded, differing mainly in the position of landmarks 5 and 10 (Figure 5A). For males, PCA also resulted in the same numbers of axes, with PC1 accounting 45.55% and PC2 for 12.67% of the variance (58.22% in total). Variation along PC1 follows a pattern similar to that observed in females. Along PC2, landmarks 2 and 8 at the positive extreme are positioned more inward, whereas landmark 5 is displaced outward, resulting in a sternum shape that is narrower anteriorly and more elongated posteriorly (Figure 5C).

Female CVA explained 56.4% of shape variation (CV1 = 34.1%; CV2 = 22.3%). *Lasiocyano sazimai* was well delimitated in the negative extreme of the axis, but partially overlapped with *Lasiodora franciscana*, *Lasiodora klugi*, *Lasiodora parahyabana*, *Lasiodora subcanens* and *Lasiodora camurujipe* (Figure 5B). Global tests confirmed significant differences among species (*p* < 0.05). Along CV1, the negative extreme of the axis is associated with a wider and more rounded sternum, reflected by the lateral position of landmarks 2 and 8 and the more proximal position of landmarks 5 and 10, resulting in a shorter and less elongated sternum. The DFA resulted in significant Procrustes distances, or Mahalanobis distances, tests between pairs of *Lasiocyano* and *Lasiodora parahybana*, *Lasiodora sertaneja* and *Lasiodora franciscana* (Table 2). Other species of *Lasiodora* paired with *Lasiocyano* reported no significative results for both distances tests. The confusion matrix from the discriminant function of these statistically significant pairs of *Lasiocyano sazimai* and *Lasiodora* presented 100% of accuracy, and cross‐validation tables had an accuracy of 68.2%, 87%, and 65.4% among pairs of *Lasiocyano sazimai* and *Lasiodora parahybana*, *Lasiodora franciscana,* and *Lasiodora sertaneja*, respectively (see Supporting Information 06).

**Table 2:** Procrustes and Mahalanobis distances between females of *Lasiocyano sazimai* and *Lasiodora franciscana*, *Lasiodora parahybana*, and *Lasiodora sertaneja*.

	*Lasiocyano sazimai*	
	Procrustes dist./*p*‐value	Mahalanobis dist./*p*‐value
*Lasiodora franciscana*	0,0259/0,083	**10,5092/0,005**
*Lasiodora parahybana*	**0,03262/0,009**	8,7001/0,09
*Lasiodora sertaneja*	**0,03868/0,009**	3,5555/0,233

*Note:* Bold values = significant *p*‐values.

For males, CVA explained 77.8% of shape variation (CV1 = 62.3%; CV2 = 15.5%). Despite partial overlap with a single individual of *Lasiodora benedeni*, males of *Lasiocyano sazimai* were separated from *Lasiodora* species along CV1, occupying its negative extreme (Figure 5D). Within *Lasiodora*, the species polygons were well‐defined and overlapped, except for *Lasiodora sertaneja*, isolated in the positive portion of CV1 axis. In *Lasiodora*, landmarks 2 and 8 show an outward displacement, corresponding to a narrower and more elongated sternum. Species differences were corroborated by the global test (Goodall's F; *p* < 0.05). The DFA resulted in significant Procrustes tests between pairs of *Lasiocyano sazimai* and *Lasiodora klugi*, *Lasiodora sertaneja* and *Lasiodora subcanens* (Table 3). Other species of *Lasiodora* paired with *Lasiocyano* reported no significative results for both distances tests. The confusion matrix from the discriminant function of these statistically significant pairs presented 100% of accuracy, and the cross‐validation tables had an accuracy of 50%, 78.9%, and 33.3% among the pairs of *Lasiocyano sazimai* and *Lasiodora klugi*, *Lasiodora sertaneja,* and *Lasiodora subcanens*, respectively (see Supporting Information 07).

**Table 3:** Procrustes and Mahalanobis distances between males of *Lasiocyano sazimai* and *Lasiodora klugi*, *Lasiodora sertaneja,* and *Lasiodora subcanens*.

	*Lasiocyano sazimai*	
	Procrustes dist./*p*‐value	Mahalanobis dist./*p*‐value
*Lasiodora klugi*	**0,03675/0,017**	76,6846/0,088
*Lasiodora sertaneja*	**0,0405/0,003**	16,1418/0,1010
*Lasiodora subcanens*	**0,0365/0,041**	16,1758/0,369

*Note:* Bold values = significant *p*‐values.

### 3.3 Outlines‐Based Results

For the Elliptical Fourier Analysis, 10 harmonics were used to describe the shape of prolateral view of palpal bulbs and dorsal view of the embolus of each sample, capturing over > 99% of shape variation. The PCA of the prolateral outline explained 59.5% of variation in the first two axes (PC1 = 42.31%; PC2 = 17.19%), with PC1 extreme shapes most related to the superior curvature of the tegulum (Figure 5E). The LDA explained 67.29% of outline variance (LD1 = 36.44%; LD2 = 30.85%), with the variation along LD1 explaining the separation of *Lasiocyano sazimai* from *Lasiodora* species at the positive portion of LD1. Considering both LD1 and LD2, five groups were recognized: (1) *Lasiocyano sazimai*, (2) *Lasiodora parahybana*, (3) *Lasiodora subcanens*, (4) *Lasiodora camurujipe*, *Lasiodora benedeni* and *Lasiodora sertaneja*, (5) *Lasiodora klugi* and *Lasiodora franciscana* (Figure 5F). In confusion matrix, the average hit rate was 100%. However, the PERMANOVA did not recovered a significant *p*‐value for species separation (*p* = 0.094). For the dorsal embolus outline PCA recovered 80.09% of variation (PC1 = 64.73%; PC2 = 15.36%), with the variation of extreme shapes of PC1 related with the proximal portion of the embolus (Figure 5G). The LDA presented 66.46% of variation in both axes (LD1 = 49.22%; LD2 = 17.24%), with *Lasiocyano sazimai* in the medium portion of LD1, without overlaps with *Lasiodora*. Despite the grouping of *Lasiodora benedeni* and *Lasiodora subcanens*, species were separated from each other, without overlaps in either axes (Figure 5H). As in the prolateral view, the confusion matrix of dorsal embolus view represented 100% of accuracy, but PERMANOVA did not recovered significant statistics to differ species (*p* = 0.093). For complete outputs of these analysis, see Supporting Information 08.

## 4 Discussion and Conclusion

The *Lasiocyano sazimai* and *Lasiodora* lineage represents a notable case of morphological similarity within Theraphosidae, in which the few characters traditionally used for diagnosis are subtle, ambiguous, and often difficult to interpret. Exploring how morphological variation is structured in systems characterized by high morphological similarity provides an important perspective on the role and limitations of morphology in species delimitation in Mygalomorphae.

In the linear analysis, *Lasiocyano sazimai* showed significant differences among females and males of several species of *Lasiodora*, suggesting interspecific divergence in the set of ratios used in analysis. Nevertheless, the disparity in accuracy before (females: 77.08%; males: 95.45%) and after cross‐validation Discriminant analysis (females: 37.5%; males: 36,36%) indicates a clear case of overfitting, in which fitting the training data too closely increases error rate on future predictions (Hastie et al. 2009). The low cross‐validated accuracy suggests that, although statistical differences exist, ratios may not be sufficiently robust or phylogenetically informative to consistently differentiate the species. This difficulty in pattern recognition and generalization is likely aggravated by the small sample size per species, a factor known to cause overfitting in Discriminant analysis and hinder stability of model prediction (Qu and Pei 2024).

In geometric analysis, *Lasiocyano sazimai* exhibited a broader, rounded sternum shape, driven by a larger inter‐landmark distance between the insertion area of left and right coxae I, in females and males, and a shorter inter‐landmark distance between the insertion of left and right coxae IV and the point of maximum median basal curvature of the labium, only in females. The Canonical Variate Analysis statistically supported the variation of the landmarks position between species of *Lasiodora* and *Lasiocyano sazimai* in both sexes; however, the males scatterplot showed a clearer separation among species polygons compared with females (Figure 5D). Nonetheless, cross‐validation tables confirmed the same predictive limitation observed in linear analysis, revealing the overfitting when distinguishing *Lasiocyano sazimai* and *Lasiodora* species pairs. Complementarily, the Procrustes tests identified significant dissimilarities in landmarks configuration between females of *Lasiocyano sazimai* and *Lasiodora parahybana* and *Lasidora sertaneja*, and between males of *Lasiocyano sazimai* and *Lasiodora klugi*, *Lasiodora sertaneja*, and *Lasiodora subcanens*. The non‐significant Mahalanobis distances for remaining species indicate high intraspecific variation, which hinders the identification of interspecific morphological patterns, resulting in extensive species overlap and low cross‐validation accuracy. Additionally, females of *Lasiocyano sazimai* and *Lasiodora franciscana* showed significant Mahalanobis distances but not significant Procrustes results. This discrepancy may reflect incomplete removal of allometric effects. The separation between size and shape is inherently challenging, and even after correction procedures, a portion of shape variation may remain associated with size (Zelditch et al. 2012; Klingenberg 2016). As a result, residual allometric variation may be incorporated into the multivariate structure of shape (Monteiro 1999) and subsequently detected by distance‐based tests, such as Mahalanobis, as apparent morphological differentiation. These results highlight the combined effects of limited sample sizes and substantial intraspecific variation, as restricted sampling does not capture the full morphological range within species, while high intraspecific variation promotes overlaps and reduces the robustness of group separation (Cardini and Elton 2007; Fonseca‐Ferreira et al. 2023).

In the recent revision of *Lasiodora*, Bertani (2023) assembled a morphological matrix in which four characters were coded as distinct for *Lasiocyano* and *Lasiodora*: male palpal bulb apical keel and male palpal tibia (discrete characters), and ratios of embolus length/width and embolus length/tegulum length (continuous characters) (Table 4). Regarding the continuous characters, values reported by Bertani—each based on a single specimen—appeared to indicate higher values in *Lasiocyano sazimai* than in *Lasiodora*, suggesting a longer and slenderer embolus in the former (Table 5). However, when additional individuals were incorporated into the data set, our linear analysis did not corroborate these differences. ANOVA revealed no significant variation among species for either ratio, and boxplots showed overlap in interquartile ranges and similar median values, indicating that these ratios vary within a largely shared range across species (Figure 4E,F). Complementarily, the density plots exhibited broad interspecific overlap and high dispersion, particularly for the embolus length and width (Figure 4G,H). These findings indicate that the apparent differences reported previously may reflect limited sampling rather than diagnostic morphological trends. Thus, the continuous characters proposed by Bertani (2023) did not provide consistent interespecific structuring to differenciate *Lasiocyano sazimai* and *Lasiodora*.

**Table 4:** Discrete and continuous characters scored with distinct states in *Lasiocyano* and *Lasiodora* spp in the morphological matrix of Bertani (2023).

	Character	State
Discrete	(2) Male palpal bulb, apical keel	(0) Short, restricted to embolus apex
		(1) Intermediate, extended slightly to the base
		(2) Long, extended to the full embolus length
	(23) Male palpal tibia (figures 5–7 in Bertani (2023))	(0) More or less straight, lacking a distal depression and a subdistal retrolateral slight projection.
		(1) With subdistal retrolateral slight projection and a distal depression
	(34) Stridulatory setae	(0) Absent(1) Present
Continuous	(0) Male bulb, embolus length by embolus width	See Table 5
	(1) Male bulb, embolus length by tegulum length	

**Table 5:** Ratios of the male palpal bulb embolus and tegulum adapted from Bertani (2023) (Table 2 in Bertani 2023). Character 0: embolus length/embolus width; Character 1: embolus length/tegulum length.

Taxon	Character 0	Character 1
*Lasiocyano sazimai*	4.22	1.56
*Lasiodora benedeni*	3.15	0.90
*Lasiodora camurujipe*	2.76	1.10
*Lasiodora franciscana*	3.21	0.95
*Lasiodora klugi*	3.15	1.12
*Lasiodora parahybana*	2.96	0.95
*Lasiodora sertaneja*	3.20	1.04
*Lasiodora subcanens*	3.44	0.92

Among the discrete characters proposed by Bertani (2023), the apical keel of the embolus is particularly problematic because its discrete states are derived from underlying continuous variation and were coded as qualitative conditions (Table 4). Discretizing continuous characters overlooks the gradual intraspecific variation inherent to these structures and introduces subjectivity, as the interpretation of states depends on viewing angle, specimen preservation, and individual morphological variation. This difficulty is particularly evident in the morphology of the male palpal bulb. Although Bertani (2000) attempted to homologize this structure and establish a standardized terminology for palpal bulb keels, the definition of character states still depends largely on qualitative descriptors with poorly defined limits. In the present case, distinguishing whether the apical keel is “short, restricted to the embolus apex” or “intermediate, slightly extending toward the base” represents an ambiguous boundary highly susceptible to observer interpretation. Such artificially discretized conditions have long been recognized as sources of erroneous species delimitations and of excessive homoplasy in phylogenetic hypotheses when continuous traits are forced into discrete classes lacking natural thresholds (Wiens 2001; Huber 2003; Sereno 2007). Although palpal bulb keels can be informative at higher taxonomic levels, their subtle variation within Theraphosidae often obscures the definition of clear character states, specially in close morphological taxa as *Lasiocyano sazimai* and *Lasiodora*. By contrast, the presence or absence of stridulatory setae—consistently present in *Lasiodora* and absent in *Lasiocyano*—constitutes a truly discrete character and remains the most reliable morphological feature distinguishing between the two genera (Figure 2B,J).

The same issue of subjectivity was also evident in the outline analysis of male palpal bulb. Most of variation captured in the prolateral view was concentrated in the upper curvature of the tegulum, whereas in dorsal view the variation was located primarily around the embolus base (Figure 5A,C). However, PERMANOVA detected no significant differences among species in either view (*p* > 0.05). For the dorsal view, the apparent variation in the embolus base was interpreted as an artifact, since the embolus and tegulum are fused structures in Mygalomorphae and do not exhibit a clear anatomical boundary (Goloboff 1995). The absence of a precise delimitation directly affects contour‐based methods: without an objective landmark for the base of the embolus, outlines become vulnerable to subjective operator interpretation, and the resulting variation reflects methodological noise rather than true biological differences. Consequently, the variation detected at the embolus base was considered non‐informative. Although the variation observed here was interpreted as primarily methodological, similar concerns regarding the diagnostic reliability of palpal bulb morphology have been raised in Theraphosidae, particularly in Aphonopelma Pocock, 1901 (Hamilton et al. 2016). Taken together, these results indicate that the overall outline of the male palpal bulb is highly similar among *Lasiodora* species and *Lasiocyano sazimai*. Thus, although outline analysis is a valuable exploratory tool for detecting subtle morphological trends, it appears less suitable as a primary diagnostic character compared with the other traits evaluated in this study.

Beyond possible morpho‐taxonomical relevance, morphological differences may also be related to ecological factors, such as climate, altitudinal, and phytophysiognomy. In terrestrial arthropods, body size and appendage proportions have been shown to response to environmental conditions, particularly temperature and humidity. In scorpions, Lira et al. (2021) proposed that the body size of *Tityus pusillus* Pocock, 1893 is increased by high temperatures and humidity of Atlantic forest. At interspecific level, the authors also discussed that the body size of *Tityus pusillus* and *Bothriurus asper* Pocock, 1893 decreases with high temperatures and in a latitudinal gradient. McCluney and Date (2008) experimented with the effects of dehydration on house crickets, in which the water‐stressed group presented a growth reduction compared to the hydrated group. For terrestrial arthropods, Horne et al. (2015) indicated the decrease and increase of body size of multivoltine (> 2 generations per year) and univoltine (one generation per year), respectively, in decreasing latitudes and warmer temperatures. Although no significant morphometric differences were detected between *Lasiodora* and *Lasiocyano* in the present study, the contrasting environmental contexts in which these taxa are recorded allow future investigations to test whether morphological variation is structured according to phytophysiognomies and whether it correlates with climatic and environmental variables. This is particularly relevant among *Lasiodora* species, some of which occur in the Atlantic Forest (*Lasiodora camurujipe*, *Lasiodora klugi,* and *Lasiodora parahybana*), whereas others are associated with forest fragments within the Cerrado (*Lasiodora franciscana* and *Lasiodora benedeni*), the Caatinga (*Lasiodora sertaneja*), and with humid forest enclaves and high‐altitude areas (“*brejos de altitude*”, in Brazilian Portuguese) in the Caatinga (*Lasiodora parahybana*) (Bertani 2023).

Taken together, these results show that even an extensive morphometric framework recovers only limited morphological structure for distinguishing *Lasiocyano sazimai* from species of *Lasiodora*. The high morphological homogeneity within these genera is evident in our results, as none of the morphometric approaches succeeded in distinguishing *Lasiocyano sazimai* from *Lasiodora* species. The few characters that remain informative for separating *Lasiocyano sazimai* from *Lasiodora* are the presence of stridulatory setae in *Lasiodora* and their absence in *Lasiocyano*, as well as the distinctive blue‐purplish setae covering the legs, carapace, and chelicerae of *Lasiocyano sazimai* (Bertani 2011; Bertani and Leal 2016; Bertani 2023). Despite the lack of diagnostic power recovered here, morphometric approaches remain valuable tools in taxonomy, as they can reveal underlying morphological structure and provide statistical support for intra‐ and interspecific delimitation (e.g., Hamilton et al. 2016; Korba et al. 2022; Colpani‐Sartori and Guadanucci 2024; Moeller et al. 2024; Sagastume‐Espinoza et al. 2024).

The taxonomy of Theraphosidae spiders is historically known as challenging, largely due to their homogeneous morphology, and, consequently, the frequent occurrence of homoplastic characters (Raven 1990; Goloboff 1995; Bertani 2001; Bond and Opell 2002; Hedin and Bond 2006; Pérez‐Miles and Perafán 2017). An effective alternative for dealing with these taxa is to integrate morphological patterns with independent sources of evidence, including molecular data, biogeography, and ecological niche modeling (Dayrat 2005; Agnarsson and Kuntner 2007; Padial et al. 2010; Schlick‐Steiner et al. 2010; Carstens et al. 2013; Cicero et al. 2021). Such integrative approaches can help resolve complex delimitations and offer more robust taxonomic solutions, as demonstrated for several studies of Mygalomorphae spiders, mostly with Theraphosidae (Hamilton et al. 2011; Montes de Oca et al. 2016; Rix et al. 2018; Korba et al. 2022; Brandt et al. 2023; Monjaraz‐Ruedas et al. 2023; Pittella et al. 2023; Nelson et al. 2024; Groppo et al. 2025; Piccinini et al. 2025). Studies of species delimitation, phylogeography, and biogeography are necessary to provide a basis to improve future research on diversification and evolutionary patterns in Mygalomorphae spiders. They are also essential for supporting conservation efforts targeting endangered species as *Lasiocyano sazimai* (Agnarsson and Kuntner 2007; Padial et al. 2010; Coates et al. 2018; ICMBio 2018; Bond et al. 2022; Sagastume‐Espinoza et al. 2024).

## Author Contributions

**Analice G. Marquezin‐Gomes:** conceptualization, data curation, formal analysis, funding acquisition, investigation, methodology, project administration, resources, software, validation, visualization, writing – original draft, writing – review and editing. **Arthur Galleti‐Lima:** conceptualization, data curation, formal analysis, funding acquisition, investigation, methodology,project administration, resources, supervision, validation, visualization, writing – original draft, writing – review and editing. **Millke J. A. Morales:** conceptualization, data curation, formal analysis,funding acquisition, investigation, methodology, resources, software, validation, writing – review and editing. **José Paulo L. Guadanucci:** conceptualization, funding acquisition, methodology, supervision, visualization, writing – review and editing, project administration.

## Ethics Statement

This study did not involve experiments with live animals or human subjects and was conducted using preserved specimens from scientific collections. Therefore, no specific ethical approval was required.

## Conflicts of Interest

The authors declare no conflicts of interest.

## Supporting information

Supporting File 1:.

Supporting File 2:.

Supporting File 3:.

Supporting File 4:.

Supporting File 5:.

Supporting File 6:.

Supporting File 7:.

Supporting File 8:.

## Data Availability

The data that support the findings of this study are available in the Supporting Information of this article.

## References

[jmor70123-bib-0001] Agnarsson, I. , and M. Kuntner . 2007. “Taxonomy in a Changing World: Seeking Solutions for a Science in Crisis.” Systematic Biology 56, no. 3: 531–539. 17562477 10.1080/10635150701424546

[jmor70123-bib-0002] Bertani, R. 2000. “Male Palpal Bulbs and Homologous Features in Theraphosinae (Araneae, Theraphosidae).” Journal of Arachnology 28, no. 1: 29–42.

[jmor70123-bib-0003] Bertani, R. 2001. “Revision, Cladistic Analysis, and Zoogeography of *Vitalius*, *Nhandu*, and *Proshapalopus*; With Notes on Other Theraphosine Genera (Araneae, Theraphosidae).” Arquivos de Zoologia 36, no. 3: 265–356.

[jmor70123-bib-0004] Bertani, R. , R. H. Nagahama , and C. S. Fukushima . 2011. “Revalidation of *Pterinopelma* Pocock 1901 With Description of a New Species and the Female of *Pterinopelma vitiosum* (Keyserling 1891) (Araneae: Theraphosidae: Theraphosinae).” Zootaxa 2814: 1–18. 10.11646/zootaxa.2814.1.1.

[jmor70123-bib-0005] Bertani, R. , and F. Leal . 2016. “A New Species of *Pterinopelma* (Araneae: Theraphosidae) From the Highlands of the State of Minas Gerais, Brazil and Description of the Male of *P. sazimai* .” Zoologia (Curitiba) 33, no. 2: e20150190–e20150199. 10.1590/S1984-4689zool-20150190.

[jmor70123-bib-0006] Bertani, R. , and J. P. L. Guadanucci . 2013. “Morphology, Evolution and Usage of Urticating Setae by Tarantulas (Araneae: Theraphosidae).” Zoologia (Curitiba) 30: 403–418.

[jmor70123-bib-0007] Bertani, R. 2023. “Taxonomic Revision and Cladistic Analysis of *Lasiodora* C. L. Koch, 1850 (Araneae, Theraphosidae) With Notes on Related Genera.” Zootaxa 5390, no. 1: 1–116. 10.11646/zootaxa.5390.1.1. 38220997

[jmor70123-bib-0008] Bonhomme, V. , S. Picq , C. Gaucherel , and J. Claude . 2014. “Momocs: Outline Analysis Using R.” Journal of Statistical Software 56: 1–24.

[jmor70123-bib-0009] Bond, J. E. , and B. D. Opell . 2002. “Phylogeny and Taxonomy of the Genera of South‐Western North American Euctenizinae Trapdoor Spiders and Their Relatives (Araneae: Mygalomorphae: Cyrtaucheniidae).” Zoological Journal of the Linnean Society 136, no. 3: 487–534. 10.1046/j.1096-3642.2002.00035.x.

[jmor70123-bib-0010] Bond, J. E. , B. E. Hendrixson , C. A. Hamilton , and M. Hedin . 2012. “A Reconsideration of the Classification of the Spider Infraorder Mygalomorphae (Arachnida: Araneae) Based on Three Nuclear Genes and Morphology.” PLoS One 7, no. 6: e38753. 10.1371/journal.pone.0038753. 22723885 PMC3378619

[jmor70123-bib-0011] Bond, J. E. , R. L. Godwin , J. D. Colby , et al. 2022. “Improving Taxonomic Practices and Enhancing Its Extensibility—An Example From Araneology.” Diversity 14, no. 1: 5. 10.3390/d14010005.

[jmor70123-bib-0012] Brandt, S. , C. Sole , R. Lyle , and C. Pirk . 2023. “Geometric Morphometric Analysis of Ocular Patterns as a Species Identifier in the South African Endemic Trapdoor Spider Genus *Stasimopus* Simon, 1892 (Araneae, Mygalomorphae, Stasimopidae).” Evolutionary Biology 50, no. 3: 350–364.

[jmor70123-bib-0013] Cardini, A. , and S. Elton . 2007. “Sample Size and Sampling Error in Geometric Morphometric Studies of Size and Shape.” Zoomorphology 126: 121–134.

[jmor70123-bib-0014] Carstens, B. C. , T. A. Pelletier , N. M. Reid , and J. D. Satler . 2013. “How to Fail at Species Delimitation.” Molecular Ecology 22, no. 17: 4369–4383. 23855767 10.1111/mec.12413

[jmor70123-bib-0015] Cicero, C. , N. A. Mason , R. A. Jiménez , D. R. Wait , C. Y. Wang‐Claypool , and R. C. Bowie . 2021. “Integrative Taxonomy and Geographic Sampling Underlie Successful Species Delimitation.” Auk 138, no. 2: ukab009.

[jmor70123-bib-0016] Coates, D. J. , M. Byrne , and C. Moritz . 2018. “Genetic Diversity and Conservation Units: Dealing With the Species Population Continuum in the Age of Genomics.” Frontiers in Ecology and Evolution 6: 165. 10.3389/fevo.2018.00165.

[jmor70123-bib-0017] Colpani‐Sartori, M. T. , and J. P. L. Guadanucci . 2024. “The Issue of Continuous Characters in the Copulatory Bulb of *Neodiplothele* Spiders: A Morpho‐Geometric Approach.” Studies on Neotropical Fauna and Environment 60, no. 3: 201–211.

[jmor70123-bib-0018] Dayrat, B. 2005. “Towards Integrative Taxonomy.” Biological Journal of the Linnean Society 85, no. 3: 407–415.

[jmor70123-bib-0019] Fonseca‐Ferreira, R. , M. J. A. Morales , L. S. Carvalho , and J. P. L. Guadanucci . 2023. “Morphometric Analysis of a Trapdoor Spider (Araneae, Idiopidae) Across Different Brazilian Biomes Reveals the Geographic Variation of Spiders From the Caatinga Biome.” Diversity 15, no. 7: 861.

[jmor70123-bib-0020] Fox, J. , and S. Weisberg . 2019. *An R Companion to Applied Regression*, 3rd ed. Sage, Thousand Oaks CA. https://www.john-fox.ca/Companion/.

[jmor70123-bib-0071] Galleti‐Lima, A. , and J. P. L. Guadanucci . 2018. “Morphology of Setae on the Coxae and Trochanters of Theraphosine Spiders (Mygalomorphae: Theraphosidae).” Journal of Arachnology 46, no. 2: 214–225.

[jmor70123-bib-0021] Galleti‐Lima, A. , and J. P. L. Guadanucci . 2019. “Comparative Morphology of Stridulating Setae of Theraphosinae (Araneae: Theraphosidae).” Zoologischer Anzeiger 283: 58–68.

[jmor70123-bib-0022] Galleti‐Lima, A. , C. A. Hamilton , L. M. Borges , and J. P. L. Guadanucci . 2023. “Phylogenomics of Lasiodoriforms: Reclassification of the South American Genus *Vitalius* Lucas, Silva and Bertani and Allied Genera (Araneae: Theraphosidae).” Frontiers in Ecology and Evolution 11, no. 1177627: 1–19. 10.3389/fevo.2023.1177627.38516293

[jmor70123-bib-0023] The GIMP Development Team . 2025. *GNU Image Manipulation Program (GIMP)*, Version 3.0.4. Community, Free Software (license GPLv3). https://gimp.org/.

[jmor70123-bib-0024] Godwin, R. L. , V. Opatova , N. L. Garrison , C. A. Hamilton , and J. E. Bond . 2018. “Phylogeny of a Cosmopolitan Family of Morphologically Conserved Trapdoor Spiders (Mygalomorphae, Ctenizidae) Using Anchored Hybrid Enrichment, With a Description of the Family, Halonoproctidae Pocock 1901.” Molecular Phylogenetics and Evolution 126: 303–313. 10.1016/j.ympev.2018.04.008. 29656103

[jmor70123-bib-0025] Goloboff, P. A. 1995. “A Revision of the South American Spiders of the Family Nemesiidae (Araneae, Mygalomorphae). Part I: Species From Peru, Chile, Argentina, and Uruguay.” Bulletin of the American Museum of Natural History Number 224: 1–189.

[jmor70123-bib-0026] Groppo, B. B. , M. J. A. Morales , T. Belintani , and J. P. L. Guadanucci . 2025. “High Diversity and Population Structure in a Widespread Tarantula *Sickius longibulbi* (Mygalomorphae: Theraphosidae).” Available at SSRN 5066687.

[jmor70123-bib-0027] Hammer, Ø. , D. A. T. Harper , and P. D. Ryan . 2001. “PAST: Paleontological Statistics Software Package for Education and Data Analysis.” Palaeontologia Eletronica 4, no. 1: 9.

[jmor70123-bib-0028] Hamilton, C. A. , D. R. Formanowicz , and J. E. Bond . 2011. “Species Delimitation and Phylogeography of *Aphonopelma hentzi* (Araneae, Mygalomorphae, Theraphosidae): Cryptic Diversity in North American Tarantulas.” PLoS One 6, no. 10: e26207. 10.1371/journal.pone.0026207. 22022570 PMC3192178

[jmor70123-bib-0029] Hamilton, C. A. , B. E. Hendrixson , and J. E. Bond . 2016. “Taxonomic Revision of the Tarantula Genus Aphonopelma Pocock, 1901 (Araneae, Mygalomorphae, Theraphosidae) Within the United States.” ZooKeys 560: 1–340. 10.3897/zookeys.560.6264. PMC4768370 27006611

[jmor70123-bib-0030] Hastie, T. , R. Tibshirani , and J. Friedman . 2009. “Boosting and Additive Trees.” In The Elements of Statistical Learning. Springer Series in Statistics. Springer. 10.1007/978-0-387-84858-7_10.

[jmor70123-bib-0031] Hedin, M. , and J. E. Bond . 2006. “Molecular Phylogenetics of the Spider Infraorder Mygalomorphae Using Nuclear rRNA Genes (18S and 28S): Conflict and Agreement With the Current System of Classification.” Molecular Phylogenetics and Evolution 41, no. 2: 454–471. 16815045 10.1016/j.ympev.2006.05.017

[jmor70123-bib-0032] Hedin, M. , S. Derkarabetian , M. J. Ramírez , C. Vink , and J. E. Bond . 2018. “Phylogenomic Reclassification of the World's Most Venomous Spiders (Mygalomorphae, Atracinae), With Implications for Venom Evolution.” Scientific Reports 8, no. 1636: 1–1637. 10.1038/s41598-018-19946-2. 29374214 PMC5785998

[jmor70123-bib-0033] Hedin, M. , S. Derkarabetian , A. Alfaro , M. J. Ramírez , and J. E. Bond . 2019. “Phylogenomic Analysis and Revised Classification of Atypoid Mygalomorph Spiders (Araneae, Mygalomorphae), With Notes on Arachnid Ultraconserved Element Loci.” PeerJ 7, no. e6864: e6864. 10.7717/peerj.6864. 31110925 PMC6501763

[jmor70123-bib-0034] Horne, C. R. , A. G. Hirst , and D. Atkinson . 2015. “Temperature‐Size Responses Match Latitudinal‐Size Clines in Arthropods, Revealing Critical Differences Between Aquatic and Terrestrial Species.” Ecology Letters 18, no. 4: 327–335. 25682961 10.1111/ele.12413

[jmor70123-bib-0035] Huber, B. A. 2003. “Rapid Evolution and Species‐Specificity of Arthropod Genitalia: Fact or Artifact?” Organisms Diversity & Evolution, 3, no. 1: 63–71.

[jmor70123-bib-0070] Instituto Chico Mendes de Conservação da Biodiversidade (ICMBio) . 2018. Livro Vermelho da Fauna Brasileira Ameaçada de Extinção: Vol. VII: Invertebrados. 1st ed.

[jmor70123-bib-0036] Klingenberg, C. P. 2011. “MorphoJ: An Integrated Software Package for Geometric Morphometrics.” Molecular Ecology Resources 11: 353–357. 10.1111/j.1755-0998.2010.02924.x. 21429143

[jmor70123-bib-0037] Klingenberg, C. P. 2016. “Size, Shape, and Form: Concepts of Allometry in Geometric Morphometrics.” Development Genes and Evolution 226, no. 3: 113–137. 27038023 10.1007/s00427-016-0539-2 PMC4896994

[jmor70123-bib-0038] Korba, J. , V. Opatova , A. Calatayud‐Mascarell , et al. 2022. “Systematics and Phylogeography of Western Mediterranean Tarantulas (Araneae: Theraphosidae).” Zoological Journal of the Linnean Society 196, no. 2: 845–884.

[jmor70123-bib-0039] Lira, A. F. A. , S. I. A. Foerster , C. M. R. Albuquerque , and G. J. B. Moura . 2021. “Contrasting Patterns at Interspecific and Intraspecific Levels in Scorpion Body Size Across a Climatic Gradient From Rainforest to Dryland Vegetation.” Zoology 146: 125908. 33657447 10.1016/j.zool.2021.125908

[jmor70123-bib-0040] Marshall, B. M. , C. T. Strine , C. S. Fukushima , P. Cardoso , M. C. Orr , and A. C. Hughes . 2022. “Searching the Web Builds Fuller Picture of Arachnid Trade.” Communications Biology 5, no. 1: 448. 10.1038/s42003-022-03374-0. 35589969 PMC9120460

[jmor70123-bib-0041] McCluney, K. E. , and R. C. Date . 2008. “The Effects of Hydration on Growth of the House Cricket, *Acheta domesticus* .” Journal of Insect Science 8, no. 1: 1–9. 10.1673/031.008.3201 PMC3061604 20302456

[jmor70123-bib-0042] Moeller, W. , A. Galleti‐Lima , and J. P. L. Guadanucci . 2024. “Morphometric Delimitation of *Pterinopelma longisternale* (Bertani, 2001) and *Pterinopelma roseum* (Mello‐Leitão, 1923) (Mygalomorphae, Theraphosidae).” Studies on Neotropical Fauna and Environment 60, no. 3: 313–322. 10.1080/01650521.2024.2430077.

[jmor70123-bib-0043] Monjaraz‐Ruedas, R. , R. W. Mendez , and M. Hedin . 2023. “Species Delimitation, Biogeography, and Natural History of Dwarf Funnel Web Spiders (Mygalomorphae, Hexurellidae, *Hexurella*) From the United States/Mexico Borderlands.” ZooKeys 1167: 109–157. 37363739 10.3897/zookeys.1167.103463 PMC10285686

[jmor70123-bib-0044] Monteiro, L. R. 1999. “Multivariate Regression Models and Geometric Morphometrics: The Search for Causal Factors in the Analysis of Shape.” Systematic Biology 48, no. 1: 192–199. 12078640 10.1080/106351599260526

[jmor70123-bib-0045] Montes de Oca, L. , G. D'Elía , and F. Pérez‐Miles . 2016. “An Integrative Approach for Species Delimitation in the Spider Genus *Grammostola* (Theraphosidae, Mygalomorphae).” Zoologica Scripta 45, no. 3: 322–333.

[jmor70123-bib-0046] Montes de Oca, L. , R. P. Indicatti , V. Opatova , M. Almeida , F. Pérez‐Miles , and J. E. Bond . 2022. “Phylogenomic Analysis, Reclassification, and Evolution of South American Nemesioid Burrowing Mygalomorph Spiders.” Molecular Phylogenetics and Evolution 168, no. 107377: 107377. 10.1016/j.ympev.2021.107377. 34954378

[jmor70123-bib-0047] Nelson, F. , N. Micaela , and S. Daniela . 2024. “An Integrative Taxonomy Approach Evaluates the Limits of the Widespread Tarantula *Plesiopelma longisternale* (Araneae: Mygalomorphae: Theraphosidae) and Reveals a New Species From Argentina.” Zoologischer Anzeiger 308: 131–143.

[jmor70123-bib-0048] Padial, J. M. , A. Miralles , I. De la Riva , and M. Vences . 2010. “The Integrative Future of Taxonomy.” Frontiers in Zoology 7, no. 1: 16. 20500846 10.1186/1742-9994-7-16 PMC2890416

[jmor70123-bib-0049] Opatova, V. , C. A. Hamilton , M. Hedin , L. M. De Oca , J. Král , and J. E. Bond . 2020. “Phylogenetic Systematics and Evolution of the Spider Infraorder Mygalomorphae Using Genomic Scale Data.” Systematic Biology 69, no. 4: 671–707. 10.1093/sysbio/syz064. 31841157

[jmor70123-bib-0050] Perafán, C. , L. Montes de Oca , and F. Pérez‐Miles . 2025. “An Integrative Study of a New Species of *Melloina* Brignoli: an Additional Support for Melloinidae New Family (Araneae: Mygalomorphae).” Journal of Natural History 59, no. 37–40: 2337–2353. 10.1080/00222933.2025.2555445.

[jmor70123-bib-0051] Pérez‐Miles, F. , and C. Perafán . 2017. “Behavior and Biology of Mygalomorphae.” In Behaviour and Ecology of Spiders: Contributions From the Neotropical Region, 29–54. Springer International Publishing.

[jmor70123-bib-0052] Piccinini, A. , M. S. Harvey , M. G. Rix , L. W. Simmons , and J. D. Wilson . 2025. “Integrative Taxonomy of Six New Species in the *Aname spicata*‐Complex (Araneae: Mygalomorphae: Anamidae) From Western Australia's Northern Jarrah Forest Subregion.” Invertebrate Systematics 39: IS25051. 10.1071/IS25051. 41292007

[jmor70123-bib-0053] Pittella, R. S. , P. G. Bassa , E. Zefa , and F. M. Bianchi . 2023. “Using the Integrative Approach to Update a Gap of One Century: Redescription and New Distribution Records of the South American tarantulas *Grammostola pulchra* (Araneae: Mygalomorphae: Theraphosidae).” Zoological Studies 62: 5. 10.6620/ZS.2023.62-05 PMC10131071 37124869

[jmor70123-bib-0054] Qu, L. , and Y. Pei . 2024. “A Comprehensive Review on Discriminant Analysis for Addressing Challenges of Class‐Level Limitations, Small Sample Size, and Robustness.” Processes 12, no. 7: 1382. 10.3390/pr12071382.

[jmor70123-bib-0055] R Core Team . 2025. *R: A Language and Environment for Statistical Computing*. R Foundation for Statistical Computing, Vienna, Austria. https://www.R-project.org/.

[jmor70123-bib-0056] Raven, R. J. 1985. “The Spider Infraorder Mygalomorphae (Araneae): Cladistics and Systematics.” Bulletin of the American Museum of Natural History 182: 1–180.

[jmor70123-bib-0057] Raven, R. J. 1990. “Comments on the Proposed Precedence of Aphonopelma Pocock, 1901.” Bulletin of zoological nomenclature 47, no. 2: 126–127.

[jmor70123-bib-0058] Rix, M. G. , J. A. Huey , S. J. B. Cooper , A. D. Austin , and M. S. Harvey . 2018. “Conservation Systematics of the Shield‐Backed Trapdoor Spiders of the Nigrum‐Group (Mygalomorphae, Idiopidae, *Idiosoma*): Integrative Taxonomy Reveals a Diverse and Threatened Fauna From South‐Western Australia.” ZooKeys 756: 1–121. 10.3897/zookeys.756.24397 PMC5956031 29773959

[jmor70123-bib-0059] Rohlf, F. J. 2015. “The Tps Series of Software.” Hystrix 26, no. 1: 9–12.

[jmor70123-bib-0060] Sagastume‐Espinoza, K. O. , L. W. Simmons , and M. S. Harvey . 2024. “Use of Geometric Morphometrics to Distinguish Trapdoor Spider Morphotypes (Mygalomorphae: Anamidae: *Proshermacha*): A Useful Tool for Mygalomorph Taxonomy.” Journal of Arachnology 52, no. 1: 31–40.

[jmor70123-bib-0061] Sereno, P. C. 2007. “Logical Basis for Morphological Characters in Phylogenetics.” Cladistics 23, no. 6: 565–587. 34905871 10.1111/j.1096-0031.2007.00161.x

[jmor70123-bib-0062] Schlick‐Steiner, B. C. , F. M. Steiner , B. Seifert , C. Stauffer , E. Christian , and R. H. Crozier . 2010. “Integrative Taxonomy: A Multisource Approach to Exploring Biodiversity.” Annual Review of Entomology 55, no. 1: 421–438. 10.1146/annurev-ento-112408-085432 19737081

[jmor70123-bib-0063] Venables, W. N. , and B. D. Ripley . 2002. *Modern Applied Statistics with S*, 4th ed. Springer. https://www.stats.ox.ac.uk/pub/MASS4/.

[jmor70123-bib-0064] Wickham, H. 2016. *ggplot2: Elegant Graphics for Data Analysis*. Springer‐Verlag. https://ggplot2.tidyverse.org.

[jmor70123-bib-0065] Wickham, H. , R. François , L. Henry , K. Müller , and D. Vaughan . 2025. “dplyr: A Grammar of Data Manipulation.” R Package Version 1.1.4. https://dplyr.tidyverse.org.

[jmor70123-bib-0066] Wiens, J. J. 2001. “Character Analysis in Morphological Phylogenetics: Problems and Solutions.” Systematic Biology 50, no. 5: 689–699. 12116939 10.1080/106351501753328811

[jmor70123-bib-0067] Wilson, J. D. , J. E. Bond , M. S. Harvey , M. J. Ramírez , and M. G. Rix . 2023. “Correlation With a Limited Set of Behavioral Niches Explains the Convergence of Somatic Morphology in Mygalomorph Spiders.” Ecology and Evolution 13, no. 1: e9706. 36636427 10.1002/ece3.9706 PMC9830016

[jmor70123-bib-0068] World Spider Catalog . 2025. *World Spider Catalog*. Version 26. Natural History Museum Bern. http://wsc.nmbe.ch. Accessed on 02 out 2025. doi: 10.24436/2.

[jmor70123-bib-0069] Zelditch, M. , D. Swiderski , and H. D. Sheets . 2012. Geometric Morphometrics for Biologists: A Primer. Academic Press.

