# Supplementary material for: Testing the Limits of Morphology: A Comprehensive Morphometric Study of the Sister Lineages Lasiocyano Galleti‐Lima, Hamilton, Borges and Guadanucci, 2023 and Lasiodora C. L. Koch, 1850 (Theraphosidae, Mygalomorphae)
Source: J Morphol. 2026 Apr 12;287(4):e70123. doi: 10.1002/jmor.70123 (PMC13071345; doi:10.1002/jmor.70123)
Supplement: Supplementary file 3 — Supporting File 3:. [file JMOR-287-e70123-s004.docx]

| Abbreviations: |  |
| --- | --- |
| Cc/Cl | Carapace length/Carapace width |
| Ec/El | Sternum length/Sternum width (interlandmark 2-8 distance) |
| FePc/FePl | Sternum length/Sternum width (interlandmark 3-7 distance) |
| PaPc/PaPl | Embolus length/Embolus width |
| TiPc/TiPl | Embolus length/Tegulum length |
| TaPc/TaPl | Femur Palp length/Femur Palp width |
| ToPc/Cl | Patella Palp length/Patella Palp width |
| FeIc/FeIl | Tibia Palp length/Tibia Palp width |
| PaIc/PaIl | Tarsus Palp length/Tarsus Palp width |
| TiIc/TiIl | Total Palp length/Carapace width |
| MeIc/MeIl | Femur Leg I length/Femur Leg I width |
| TaIc/TaIl | Patella Leg I length/Patella Leg I width |
| ToIc/Cl | Tibia Leg I length/Tibia Leg I width |
| FeIIc/FeIIl | Metatarsus Leg I length/Metatarsus Leg I width |
| PaIIc/PaIIl | Tarsus Leg I length/Tarsus Leg I width |
| TiIIc/TiIIl | Total Leg I length/Carapace width |
| MeIIc/MeIIl | Femur Leg II length/Femur Leg II width |
| TaIIc/TaIIl | Patella Leg II length/Patella Leg II width |
| ToIIc/Cl | Tibia Leg II length/Tibia Leg II width |
| FeIIIc/FeIIIl | Metatarsus Leg II length/Metatarsus Leg II width |
| PaIIIc/PaIIIl | Tarsus Leg II length/Tarsus Leg II width |
| TiIIIc/TiIIIl | Total Leg II length/Carapace width |
| MeIIIc/MeIIIl | Femur Leg III length/Femur Leg III width |
| TaIIIc/TaIIIl | Patella Leg III length/Patella Leg III width |
| ToIIIc/Cl | Tibia Leg III length/Tibia Leg III width |
| FeIVc/FeIVl | Metatarsus Leg III length/Metatarsus Leg III width |
| PaIVc/PaIVl | Tarsus Leg III length/Tarsus Leg III width |
| TiIVc/TiIVl | Total Leg III length/Carapace width |
| MeIVc/MeIVl | Femur Leg IV length/Femur Leg IV width |
| TaIVc/TaIVl | Patella Leg IV length/Patella Leg IV width |
| ToIVc/Cl | Tibia Leg IVength/Tibia Leg IV width |
| Cc/Cl | Metatarsus Leg IV length/Metatarsus Leg IV width |
| Ec/El | Tarsus Leg IV length/Tarsus Leg IV width |
| FePc/FePl | Total Leg IV length/Carapace width |


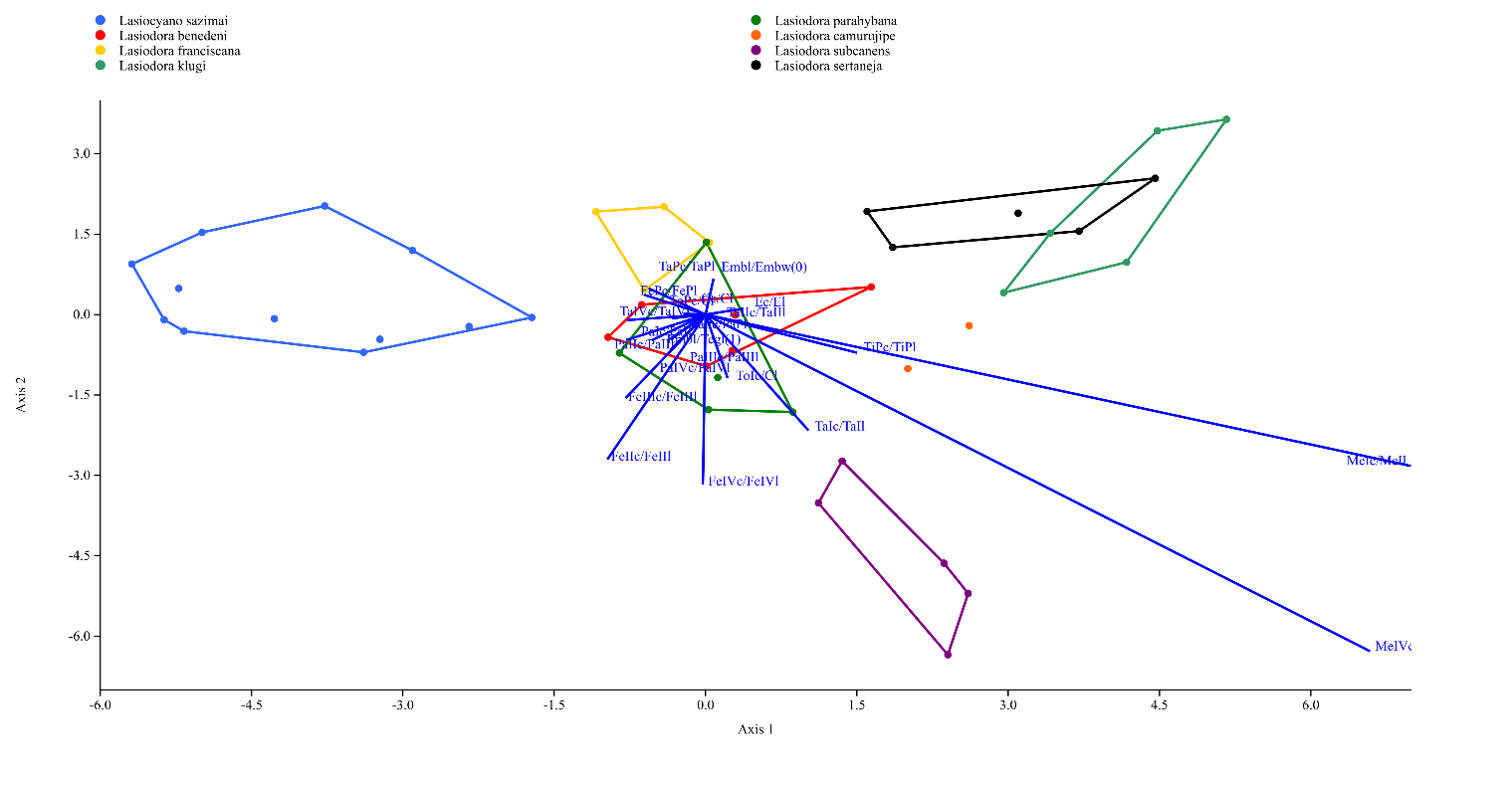
Figure 1. Linear Discriminant analysis (LDA). Males biplot.


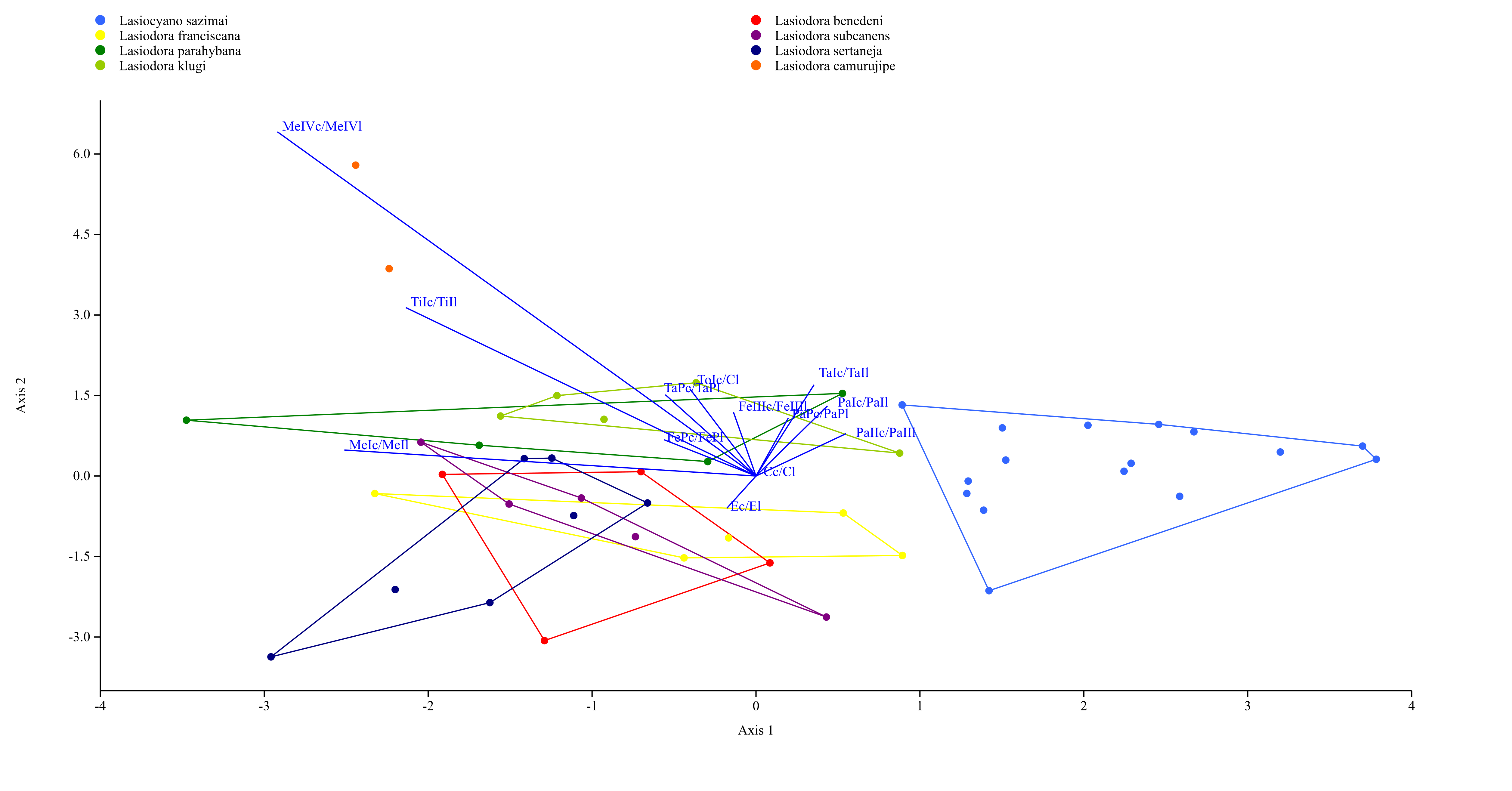


Figure 2. Linear Discriminant analysis (LDA). Females biplot.
